# The Effect of Serotonin Transmission on Depressive and Insomnia Symptoms in Inflammatory Bowel Diseases

**DOI:** 10.3390/jcm12196353

**Published:** 2023-10-04

**Authors:** Marcin Sochal, Alicja Witkowska, Agata Binienda, Agata Gabryelska, Piotr Białasiewicz, Jakub Fichna, Renata Talar-Wojnarowska, Ewa Małecka-Wojciesko

**Affiliations:** 1 Department of Sleep Medicine and Metabolic Disorders, Medical University of Lodz, 92-215 Lodz, Poland; alicja.witkowska1@stud.umed.lodz.pl (A.W.); agata.gabryelska@gmail.com (A.G.); piotr.bialasiewicz@umed.lodz.pl (P.B.); 2 Department of Biochemistry, Medical University of Lodz, 92-215 Lodz, Poland; agata.binienda@gmail.com (A.B.); jakub.fichna@umed.lodz.pl (J.F.); 3 Department of Digestive Tract Diseases, Medical University of Lodz, 92-215 Lodz, Poland; r-wojnarowska@wp.pl (R.T.-W.); ewuncia@poczta.onet.pl (E.M.-W.)

**Keywords:** IBD, serotonin, SERT, anti-TNF therapy, insomnia, depression

## Abstract

The serotonergic pathway may impact the pathogenesis and the course of inflammatory bowel diseases (IBDs). The aim of this study was to investigate the relationship between 5-HT, the serotonin transporter (SERT), and the clinical course of the disease with the occurrence of sleep and mood disorders. Participants completed sleep questionnaires and the Beck Depression Inventory (BDI). Serum 5-HT, SERT protein expression, and mRNA levels were quantified. Additionally, patients treated with anti-TNF therapy were examined before and after treatment. In this study, 77 patients with IBD and 41 healthy controls (HCs) were enrolled and 24 of them were treated with anti-TNF therapy. Patients with IBD had higher 5-HT levels and SERT protein expression than the HCs, but not mRNA SERT levels (*p* = 0.015, *p* = 0.001, *p* = 0.069, respectively). Similar results were obtained for patients in the active state of the disease compared to the non-active state. There was a positive relationship between insomnia severity and SERT protein expression. BDI did not correlate with serotonin or SERT. After anti-TNF therapy, only 5-HT levels were decreased. 5-HT and SERT protein are overexpressed in active IBD and may represent a candidate for novel disease activity biomarkers. The correlation between the SERT protein level and the severity of insomnia symptoms might be among the underlying biochemical factors of sleep disturbances. Anti-TNF treatment might contribute to the reduction in 5-HT levels.

## 1. Introduction

Inflammatory bowel diseases (IBDs) are multifactorial digestive diseases that include ulcerative colitis (UC) and Crohn’s disease (CD). They are characterized by chronic, relapsing–remitting inflammation of the digestive tract [1]. The etiology of IBD is not fully understood. Nevertheless, gut dysbiosis, dysregulation of the immunological system, and intestinal barrier impairment were identified as risk factors contributing to the development of IBD [2,3]. Additionally, researchers have also designated stress, depression, and sleep disturbances as co-occurring conditions that may even be considered as risk factors for the development or relapse of IBD [4,5]. Mood disorders and diminished quality of life in IBD might be attributable to brain–gut axis disbalance (BGA).

BGA plays an essential role in psychological, immunological, and neurological modulation. It relies on the interactions between the peripheral and central nervous system (CNS) and intestinal response, including microbiota functions. BGA is also suggested to be of significance to sleep regulation, and sleep disorders are oftentimes comorbid with intestinal microbiota disbalance. Therefore, insomnia and altered microbiota composition are believed to coexist bidirectionally. Evidence of this may include, among other things, the fact that IL-6 plasma levels increase simultaneously with sleep loss and intestinal inflammation leads to IL-6 overexpression. According to Smith et al., IL-6 concentration is positively correlated with gut microbiota diversity, total sleep time, and time spent in bed [6,7]. Furthermore, communication within the BGA takes place via the neuroendocrine and neuroimmune systems and is mediated by the vagal nerve and neurotransmitters such as serotonin. 

Neurotransmitters are messengers that transfer signals between neurons, and from neurons to designated targets, such as muscles and endocrine tissues. Serotonin, 5-hydroxytryptamine (5-HT), has an impact on CNS and enteric nervous system (ENS) development; specifically, it has a role in governing neuronal differentiation and migration, as well as influencing axonal outgrowth, myelination, and the formation of synapses. It is also of importance to preserving mood stability, cognitive functions, immunity, and GI motility [8]. According to the available data, 90% of 5-HT is synthesized from tryptophan in enterochromaffin cells in the GI tract, whereas only 10% of systemic 5-HT comes from serotonergic neurons. Furthermore, 5-HT is converted into melatonin in the pineal gland through the usage of two enzymes: serotonin N-acetyltransferase (NAT) and hydroxyindole-O-methyltransferase (HIOMT). Additionally, melatonin is produced by the neuro-endocrine cells of the GI tract mucosa, which is of importance to the circadian rhythm and sleep regulation [9,10,11]. The serotonin transporter (SERT), encoded by the *SLC6A4* gene, regulates the extracellular concentration of 5-HT by inactivating and transporting it to mucosal enterochromaffin cells [12]. 

Sleep disturbances and mood disorders are frequently overlooked conditions accompanying IBD signs and symptoms [13]. They might result from elevated levels TNF-a and IL-1 [14]. Additionally, disruptions in circadian rhythms were proven to alter colonic motility and neurotransmitter concentration, whereas sleep disturbances have been found to unfavorably impact the GI system, causing alterations in the gut microbiota [10,15]. It was also acknowledged that IBD flare-ups can induce sleep disorders [14]. 

These findings are in line with our previous studies in which we documented that patients with IBD experience lowered sleep quality, higher daytime sleepiness, and prolonged latency compared to healthy volunteers [16]. 

Moreover, depression also often accompanies IBD. Consequently, depression and anxiety are considered to induce inflammation and the dysregulation of T-cell functions. At the same time, patients with IBD are more likely to manifest depressive symptoms [17]. Foregoing studies have found that the prevalence of depression and anxiety disorders in IBD patients is 15% and 20%, respectively; in particular, 40% of participants with CD suffer from depression [5]. In another paper, it was observed that women with CD aged 50–70 are more likely to suffer from depression and anxiety disorders [18]. Additionally, the most recent metanalysis conducted by Bisgaard et al. reports that the prevalence of depression is as high as 3.6–26.9/1000 people per year in patients with IBD, as opposed to the reference group with 2.5–12.2/1000 people per year. Similarly, anxiety was examined in 4.0–25.0/1000 people per year in individuals with IBD juxtaposed with 3.0–16.3/1000 people per year in the reference group [19].

Tumor necrosis factor (TNF) is a proinflammatory cytokine that induces and maintains intestinal inflammation [20]. Hence, blocking this pathway through the use of anti-TNF monoclonal antibodies (mAbs) is becoming one of the most widely used interventions in severe IBD treatment. Their efficacy and safety have been proven in recent years [21]. Concurrently, 5-HT may also modulate the peripheral immune system response by being stored and transported in peripheral immune cells such as macrophages, and dendritic cells. Additionally, 5-HT contributes to T-cell activation and is re-uptaken by B-cells with 5-HTR3A receptors [22].

The effect of TNF-alpha antibodies on 5-HT action has rarely been studied. In particular, the role of 5-HT, SERT, in relation to sleep disturbances in IBD has not been well elucidated yet. Therefore, this study aimed at exploring the correlation between 5-HT and its transporter levels in regard to IBD flare-ups, and concomitant sleep and mood disorders. Furthermore, the possible effect of anti-TNF treatment on previously mentioned parameters was evaluated. 

## 2. Materials and Methods

### 2.1. Study Design

This prospective study was designed in accordance with the declaration of Helsinki, conducted at the Department of Digestive Tract Diseases, Medical University of Lodz, Poland, and approved by the Ethical Committee of the Medical University of Lodz, Poland, with the following reference number: RNN/433/18/KE. 

### 2.2. Inclusion and Exclusion Criteria

The inclusion criteria for participants with IBD and HC comprised giving informed consent for participation in the study, being aged between 18 and 65 years. The IBD group was created based on previously established clinical, radiological, endoscopic, or histological diagnoses [23]. The exclusion criteria for both groups were as follows: addiction to alcohol or other psychoactive substances, psychiatric illness developed over the course of this study, abdominal surgery performed in the six months prior to the research, chronic inflammatory diseases (e.g., systemic lupus, erythematosus, rheumatoid arthritis, and chronic pancreatitis), and present or past malignant neoplastic disease.

### 2.3. Assessment of Disease Severity and Questionnaire Variables

Based on the Harvey–Bradshaw Index (HBI) scale and Partial Mayo Score (PMS) Index, patients were subdivided into subgroups: all who received 5 points or more in the HBI questionnaire were assigned to the CD-active (AC) group, and those who scored more than 2 points in the PMS were classified as the UC-A group [24,25]. The remaining patients were included in the nonactive (NA) group.

Patients signed an informed consent form and were provided with all necessary information about the study. All of the qualified participants had 4 mL of their venous blood drawn. Twenty-four patients in the state of active disease qualified for biological treatment with anti-TNF infliximab (IFX) or adalimumab (ADA) medication. In this subgroup, the blood was drawn twice: firstly, before biological treatment, and secondly, after 14-weeks. All samples were centrifuged and the obtained serum was stored at −80 °C.

Data, with particular reference to the course of the disease, the year of the diagnosis, the number of stools, and the presence of fecal blood on the day prior to examination, were collected. The occurrence of past and current complications was also considered. Patients completed various questionnaires estimating their sleep quality and mood. In regard to evaluating an individual’s quality of sleep, the Pittsburgh Sleep Quality Index (PSQI), Athens Insomnia Scale (AIS), and Epworth sleepiness scale (ESS) were used. PSQI measures subjective sleep quality. It surveys parameters such as difficulty falling asleep and maintaining sleep continuity, and the ability to function throughout the day, and explores the causes leading to sleep disorders. Therefore, PSQI was used to evaluate time in bed (time from going to bed to getting up), sleep latency (time from going to bed to falling asleep), sleep time, and sleep efficiency (sleep time/time in bed) [26]. AIS pertains specifically to the occurrence of difficulty falling asleep, its frequency, and its impact on individuals’ daily functioning. It was used to evaluate sleep efficiency [27]. With the intention of measuring daytime sleepiness, ESS was scrutinized. Patients approximated their chance of falling asleep whilst undergoing day-to-day life situations [28].

Depressive symptoms were estimated using the self-reported Beck Depression Inventory (BDI), which is mainly used to assess the severity of depressive symptoms. The cutoff points for a mild depression were estimated for outcomes greater than or equal to 11; however, the score was not indicative of a depression diagnosis [29]. 

### 2.4. Anti-TNF Therapy

Patients who had found themselves in a state of AC illness and met the additional insurers’ requirements underwent biological treatment with anti-TNF infliximab (IFX) or adalimumab (ADA). IFX was administered intravenously at a dose of 5 mg/kg body weight. The first dose was injected on admission, the second after 2 weeks, and the following after 6 weeks since the first injection. Subsequent doses were administered every 8 weeks. ADA was used subcutaneously; the first dose was 160 mg, the second 80 mg, and the third 40 mg. Notwithstanding that IFX and ADA are differentiated by their means of administration, they are both found to be equally effective in treating CD and UC according to the European Crohn’s and Colitis Organization (ECCO). This subgroup was examined twice: firstly, before inducing the treatment plan, and secondly after the completion of the 14-week therapy plan. 

### 2.5. Evaluation of Gene Expression and Protein Concentration

The serum levels of 5-HT and SERT were assayed at the Department of Sleep Medicine and Metabolic Disorders, Medical University of Lodz, Poland, using the enzyme-linked immunosorbent assay (ELISA), a validated method. 5-HT was determined via a Dermeditec test (Kiel, Germany; standard range: 15–2500 ng/mL, serum value: 20 μL) and SERT via an Elab test (Nanjing, China; standard range: 31.2–2000 pg/mL, serum value: 1 μL/100 μL sample). Absorbance was assessed using a 450 nm spectrum via an 800TS Absorbance Reader (Agilent Technologies, Santa Clara, CA, USA). 

A quantitative real-time polymerase reaction was executed at the Department of Biochemistry, Medical University of Lodz, Poland. The integral genetic material was RNA obtained via isolation from peripheral blood mononuclear cells (PBMC) by means of the trizol (Invitrogen) method. The RNA’s Integrity Number (RIN) and conentration of the isolated RNA were measured using a Picodrop Microliter UV/Vis spectrophotometer (Picodrop Limited, Hinxton, Cambridgeshire, UK). Reverse transcription was performed on a dedicated kit from the manufacturer (Maxima First Strand cDNA Synthesis Kit, Thermo Fisher Scientific Inc., CA, USA). Then, a reverse-transcription polymeraze chain reaction was performed. The mixture reaction consisted of cDNA, nuclease-free water, and gene-specific probes (TaqMan assays for SERT and referencing gene: beta-actin). The cycle threshold (CT) was computed and modified into ΔCT. Gene expression was assessed by means of the Livak method [30].

### 2.6. Statistical Analysis

Statistica 13.1PL (StatSoft, Tulsa, OK, USA) was used for data analysis. The threshold of statistical significance was set to *p* < 0.05. The Shapiro–Wilk test was applied to estimate the normality of data distribution, and the variables were presented in the form of standard deviation. Otherwise, the median and interquartile range (IQR) were computed. Differences between nominal variables were examined depending on the strength of the least abundant group (n_min_) viaChi^2^ (n_min_ ≥ 10) and Fisher’s exact test (n_min_ < 10). A Mann–Whitney U test was used for analyzing independent continuous variables for an abnormal distribution. Moreover, the Wilcoxon test was of assistance in comparing dependent variables before and after treatment in an abnormal distribution. Assessments of the correlations between variables were executed using Spearman’s rank correlation test.

## 3. Results

In total, 118 patients were enrolled, of whom 77 were constantly treated for IBD (among whom 46 had UC and 31 had CD) and 41 healthy controls. The demographic and clinical data are presented in Table 1. The IBD and HC groups were matched regarding patients’ age, sex, and BMI. 

In general, individuals with IBD experienced prolonged sleep latency, had lower sleep efficiency, and had more severe depression symptoms than the HCs. Additionally, 19 participants with IBD (24.6%) and 6 HCs (14.6%) had mild depression, as diagnosed via the BDI (*p* = 0.301). Patients in the AC group obtained higher results than the HCs in all the sleep questionnaires, excluding ESS. Sleep time in the AC group remained similar to that in the HCs. Furthermore, the NA group spent more time in bed and their sleep efficiency was markedly lower than the HCs (Table 1).

Patients with CD had higher scores than the HCs in all questionnaires, excluding ESS and PSQI. On the other hand, patients with UC attained higher results than the HCs only in the BDI scale. The results of individuals with CD in AC did not differ from those in the NA group, and the only difference between participants with UC in the AC and NA groups was observed in sleep time (Table 2).

At the first time point, the mean serum level of 5-HT and SERT protein expression was significantly higher in patients with IBD compared to the HCs (*p* = 0.015 and *p* = 0.001, respectively). 5-HT and SERT protein concentrations were also elevated in the AC compared to the NA group (*p* = 0.001 and *p* < 0.001, respectively) and in the AC vs. the NA group (*p* = 0.014 and *p* = 0.021, respectively). Differences in mRNA *SERT* levels were not observed between the groups (Table 1).

No differences in the mean 5-HT and SERT protein concentration between the UC and CD groups (*p* = 0.251 and *p* = 0.628, respectively) were observed. Patients with UC but not CD had higher serum levels of 5-HT compared to the HCs (*p* = 0.008 and *p* = 0.091, respectively). Serum SERT protein expression was higher in both individuals with UC and CD compared to HC (*p* < 0.001 and *p* = 0.005, respectively). In UC, the mean serum level of 5-HT was similar in the AC and NA groups (*p* = 0.764), while SERT protein concentration was higher in AC than in NA group (*p* = 0.025). On the other hand, in the CD group, mean serum 5-HT in AC was higher in AC than NA (*p* = 0.002), while SERT protein expression in the AC and NA groups was similar (*p* = 0.197). The mRNA *SERT* level was similar in each examined group (Table 2).

There were positive correlations between 5-HT level in patients with IBD and HBI but not between PMS scores (R = 0.44; *p* = 0.002; *p* > 0.05, respectively). No connections were observed between age, BMI, and 5-HT in either IBD or HC. SERT protein expression did positively correlate with the serum level of 5-HT in the HCs (R = 0.43; *p* = 0.005), but not in the IBD group (R = 0.11; *p* = 0.357). In addition, no correlation was found between SERT protein concentration and age, BMI, HBI, or PMS. mRNA *SERT* level did not correlate with either 5-HT, SERT protein expression, or baseline characteristics and sleep questionnaires values (Table 3).

Factors such as sex, smoking, history of surgery, and other chronic diseases did not correlate with the serum concentration of 5-HT and SERT protein expression in the IBD group. Additionally, most of the sleep questionnaires’ outcomes were not associated with 5-HT level and SERT protein concentration. The only positive correlation was noted in the AIS scale, i.e., patients who obtained more than five points on the AIS scale had significantly higher SERT protein expression (*p* = 0.006) compared to patients who received more than 5 points on the same scale. IBD men had higher mRNA *SERT* levels in comparison to women (*p* = 0.011). The same difference was observed between nonsmokers and smokers (*p* = 0.049) in the IBD group. History of surgery, as well as other chronic diseases, was not associated with mRNA *SERT* level. Moreover, mRNA *SERT* level was not correlated with BDI, AIS, ESS, or PSQI results (Table 4).

**Figure 1:**
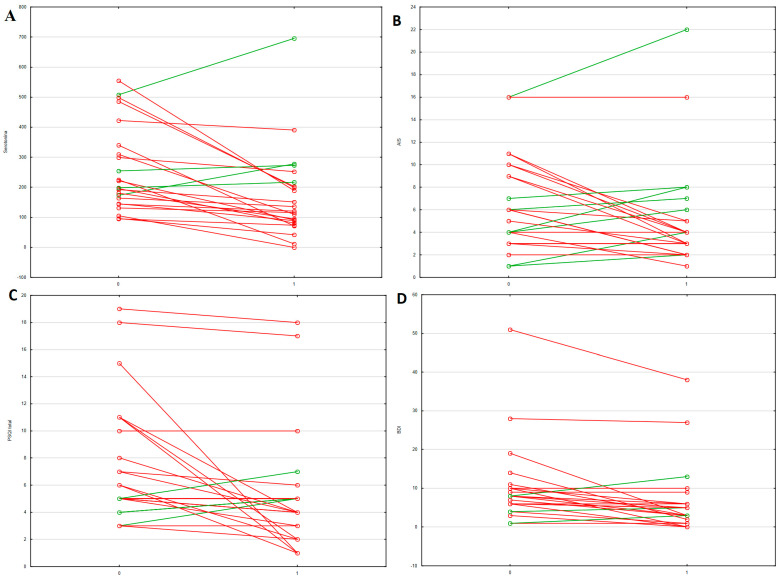
Changes in selected parameters in patients with IBD before and after completing therapy: (**A**) serum level of serotonin, (**B**) AIS, (**C**) PSQI, (**D**) BDI. PSQI: Pittsburgh Sleep Quality Index, BDI: Beck Depression Inventory, AIS: Athens Insomnia Scale. Red line—decrease parameter; green line—increase parameter.

After 14 weeks of biological therapy, a notable decrease in 5-HT level was observed (198.2 IQR: 145.1–309.8 vs. 128.6 IQR: 85.0–209.9 ng/mL; *p* = 0.001) in 24 patients, but no changes were observed in SERT level (19.8 ± 9.6 vs. 20.1 ± 9.2 ng/mL; *p* = 0.865) and mRNA SERT level (1.5 IQR: 0.7–2.9 vs. 1.2 IQR: 0.5–2.4; *p* = 0.732). In addition, after 14 weeks, participants with IBD obtained lower scores in the BDI (8 IQR: 6–10 vs. 3 IQR: 3–6; *p* = 0.002), AIS (6 IQR: 4–10 vs. 4 IQR: 3–5; *p* = 0.044), and PSQI (6 IQR: 5–11 vs. 4 IQR: 3–5; *p* = 0.005) questionnaires, but not in the ESS (7.2 ± 3.7 vs. 5.7 ± 4.1; *p* = 0.064). The results are presented in the form of Figure 1.

## 4. Discussion

The dysregulation of SERT is extrapolated in profuse digestive disorders, but there is a scarcity of data covering the subject of serotonergic pathways in IBD [31].

In this study, the 5-HT level and SERT protein concentration were significantly higher in the IBD group than in the HCs, whereas the mRNA *SERT* level did not differ statistically significantly in both the IBD and HC group. The elevation of the serum level of 5-HT in IBD is consistent with previous research [32,33]. Wang et al. observed that a greater Pouchitis Disease Activity Index (PDAI) upon endoscopy, which representsthe worst clinical state, was associated with a higher serum level of 5-HT [34]. Moreover, in a 2,4,6-trinitrobenzenesulfonic acid (TNBS)-induced model of guinea pig ileitis, O’Hara et al. observed an increase in the number of 5-HT-immunoreactive cells in the inflamed mucosa [35]. The study also reported a reduction in SERT immunoreactivity in the epithelium, which is not consistent with our research [35]. Jorandli et al. showed that mRNA *SERT* level is reduced in the inflamed ileal and colonic areas of patients with CD ileitis, CD colitis, and UC colitis in comparison with HCs [36]. On the other hand, our study was based on the peripheral SERT protein concentration and mRNA *SERT* level; their levels might be attributable to different regulatory mechanisms to the ones present in the intestinal epithelium. Nevertheless, to date, the available data on this subject are scarce.

The obtained data might be explained by the proinflammatory properties of the serotonergic pathway. 5-HT can influence the production of reactive oxygen species in the colon and can function solely as an inflammatory mediator, whilst activating the 5-HT7 receptor [37]. According to Qasem et al., *Mycobacteria paratuberculosis* infection in participants with CD caused the activation of Toll-like receptor 2 (TLR-2) and the production of pro-inflammatory cytokines, and resulted in SERT inhibition [38]. It is unclear whether the changes in the serotonergic pathway were the root of the digestive tract inflammation or were present as its consequence. 

In our study, the 5-HT level was elevated in patients with CD in the AC compared to the NA group, as opposed to the UC group, where a flare-up of the disease did not correlate with the 5-HT concentration. Our results in the CD group are consistent with previous studies; the same association was observed between CD severity and serum level of 5-HT [33]. In our study, in the CD group, the SERT protein expression was not changed during a disease flare-up, but in the UC group, a higher SERT protein concentration was observed in EX compared to RE. Manzella et al. indicated that individuals with CD with mild and severe inflammation had lower SERT immunoreactivity in both ileal and colonic samples compared to HCs, which might also be associated with a higher serum level of 5-HT. Because of the insufficient SERT protein concentration on the epithelial cells, the increase in the extracellular levels of 5-HT resulted in an increase in the amount of circulating 5-HT that could be taken up by platelets [33].

Additionally, in our study, a positive correlation between mRNA *SERT* level and smoking was observed. Smoking is thought to have an impact on the 5-HT level and platelet aggregation. It was shown that the basal serum level of 5-HT is higher in smokers than non-smokers. Moreover, smokers re-examined 15 min after nicotine exposure had increased serum levels of 5-HT compared to their serum levels 4 h after smoking [39]. The reason for this observed relationship may be the fact that smoking activates the sympathetic nervous system and stimulates the secretion of 5-HT from enterochromaffin cells [40]. Another study, on an animal model, focused on the effect of nicotine on mRNA *SERT* levels in the raphe nuclei of rats and demonstrated downregulation of the receptors and a decrease in mRNA *SERT* levels, triggered by chronic nicotine exposure [41]. Cigarette smoking is thought to have an impact on CD progression, but it is unimpactful toward UC progression, or may even have a beneficial effect in UC flare-ups, as well as reducing the need for colectomy [42]. Nicotine mediates the α7 nicotinic receptors present in the immune cells, and in UC, it downregulates the production of proinflammatory cytokines; the CO present in cigarette smoke impairs dendric cell function and antigen presentation and mitigates intestinal inflammation. On the other hand, CD is associated with increased oxidative stress; smoke and its constituents exacerbate oxidative damage and therefore might worsen general conditions in patients with CD [43]. Overall, the observed differences might be attributable to the dissimilar pathogeneses of the diseases. There might be an underlying connection between the higher serum level of 5-HT in UC and insensitivity to the effects of smoking on the diseases’ pathogenesis. 

Another plausible explanation for the differences observed between the functioning of the serotonergic pathway in CD and UC may be attributed to gender. Men with IBD had higher mRNA *SERT* levels than women; however, 5-HT levels and SERT protein expression remained similar in both IBD males and females. These results might be attributable to the influence of sex hormones such as estrogen on the serotonergic pathway. Based on a spayed monkey model, estrogen exposure had an impact on the downregulation of mRNA *SERT* levels. On the other hand, prolonged estrogen treatment increased mRNA *SERT* levels, whereas progesterone did not have any impact on them [44]. Furthermore, it is known that predisposition to developing IBD can be impacted by mutations in susceptible loci on the long arm of the X chromosome; therefore, female gender may play a role in the disease’s prevalence and severity [45]. Since there might be a connection between sex and 5-HT levels, further research would be beneficial to understanding the underlying mechanisms of the estrogen–serotonergic pathway in IBD. 

After 14 weeks of anti-TNF therapy, a notable decrease in 5-HT level was observed without any changes in SERT protein expression and mRNA *SERT* level. Additionally, the results of the BDI, AIS, and PSQI questionnaires showed improvement. To the best of our knowledge, there are no similar studies focusing on the impact of biological therapy on the serotonergic pathway in IBD. However, similar results were obtained in other inflammatory diseases, namely, ankylosing spondylitis (AS) and rheumatoid arthritis (RA). It was observed that patients with AS had lower baseline levels of 5-HT compared to those with RA and the HCs. The serum level of 5-HT in AS further decreased because of the instigated anti-TNF treatment [46]. Therefore, 5-HT might be regarded as a factor contributing to the development of IBD symptoms, and the concentration of 5-HT can indicate the severity of the disease. It is conceivably possible that there is an interaction between 5-HT and TNF. Nevertheless, the exact mechanisms of this possible relationship have not been elucidated yet.

Additionally, the serotonergic pathway was proven to be linked to depressive disorder [47]. Our results are consistent with Barberio et al., who confirmed that depression frequently co-occurs with IBD, particularly in disease flare-ups [48]. Despite the fact that 5-HT is of crucial importance to the pathogenesis of depressive disorder, we did not observe any relationship between BDI, 5-HT, SERT protein, and mRNA *SERT.* This lack of association could result from the high subjectivity of the BDI questionnaire, which is filled out by a patient, not a clinician. 

Depressive disorders are oftentimes associated with diminished sleep quality. According to our present and previous studies, patients with IBD have significantly lower sleep quality than HCs [17,49]. The elevated SERT protein, but not 5-HT and mRNA *SERT* levels, was associated with the higher AIS questionnaire outcomes. Nevertheless, these observations cannot be explained since there are no relevant human and animal studies available. It was only established that the downregulation of *SERT* expression was associated with functional alterations in 5-HT neuro-transmission, as shown by the increased capacity of the 5-HT1A receptor in adult rats [50].

This study has several limitations. Sleep quality was assessed subjectively using questionnaires. However, the most common sleep disturbance—insomnia—was diagnosed based on medical history, sleep diaries, and questionnaires, which are commonly accepted, validated, and used worldwide. Objective diagnostic study methods such as polysomnography are applied to exclude other causes of diminished sleep quality than insomnia, and do not allow for the assessment of the chronicity of the disorder [51]. The BDI questionnaires were completed by patients based on their subjective evaluations of depression. Patients’ insights might not have been as objective as the clinical diagnosis of the depressive disorders’ intensity. A further limitation of this study is the difficulty in the clinical measurement of the severity of IBD. It was not possible to conclusively determine whether patients were in the AC or NA stage of the disease, since the data from endoscopic examinations presented by the patients were incomplete. Therefore, the 5-HT, SERT, and mRNA *SERT* levels were examined in serum and leukocytes. Additionally, it has been reported that tryptophan metabolism is altered in IBD, especially in the active state of the disease, which needs to be considered in further research [52]. Furthermore, the SERT gene polymorphisms, and therefore, altered 5-HT reuptake might have impacted the obtained results. Nevertheless, the study group was too small to evaluate it. 

## 5. Conclusions

The differences in the levels of 5-HT and SERT protein between CD and UC indicate potential variations in the pathogenesis of these diseases. Considering that SERT protein expression was correlated with the intensity of insomnia, but not depressive symptoms, it might be the basis for biochemical sleep disturbances, commonly occurring in IBD. mRNA *SERT* level appears to be unimpactful toward IBD symptoms. 5-HT and SERT protein are overexpressed in IBD flare-ups. Additionally, anti-TNF therapy seems to be effective in reducing the serum level of 5-HT and appears to accompany improvements in depressive symptoms and sleep quality. Further research would be beneficial to better understand the underlying biochemical and molecular mechanisms of sleep disorders in IBD. A detailed understanding of the serotonergic pathway is crucial to improving psychological and somatic well-being; therefore, gaining knowledge about this pathway can significantly influence the future clinical management of IBD.

## Figures and Tables

**Table 1:** Participants’ characteristics, the results of sleep- and mood-related questionnaires, and quantification of mRNA SERT and serotonin levels and SERT protein expression in patients with IBD subcategorized into A and NA groups.

	IBD (n = 77)	AC (n = 45)	NA (n = 32)	HC (n = 41)	*p* (IBD-HC)	*p* (AC-HC)	*p* (NA-HC)	*p* (AC-NA)
Women (n, %)	43, 55.8	24, 53.3	19, 59.4	20, 48.8	0.464	0.673	0.368	0.599
Age	35 (28–41)	35 (31–41)	37 (25–42)	31 (25–44)	0.518	0.288	0.929	0.518
BMI	23.3 (20.8–25.9)	24.3 (±3.2)	22.9 (±3.2)	23.8 (±3.5)	0.765	0.515	0.285	0.094
Smoker (n, %)	11, 14.3	6, 13.3	5, 15.6	4, 9.8	0.572	0.741	0.493	1.000
Chronic diseases (n, %)	18, 23.4	15, 33.3	3, 9.4	5, 12.2	0.222	0.024	1.000	0.016
Immunomodulators (n, %)	27, 35.1	14, 31.1	13, 40.6	-	-	-	-	0.389
mRNA SERT	1.3 (0.6–2.2)	1.2 (0.7–2.2)	1.4 (0.5–2.0)	1.7 (1.0–2.3)	0.069	0.117	0.121	0.971
Serotonin	145.1 (100.0–192.4)	164.1 (105.4–221.0)	130.0 (87.8–165.7)	115.3 (76.6–160.5)	**0.015**	**0.001**	0.480	**0.014**
SERT	18.6 (±8.8)	20.5 (±9.4)	15.9 (±7.1)	13.2 (±6.0)	**<0.001**	**<0.001**	0.079	**0.021**
BDI	7 (4–10)	7 (5–10)	6.5 (3.0–10.5)	3 (1–8)	**0.005**	**0.003**	0.101	0.268
AIS total	5 (4–9)	6 (4–9)	5 (3–7)	4 (2–7)	0.052	**0.021**	0.393	0.156
ESS	6 (4–10)	6 (5–10)	5 (3–9)	6 (3–8)	0.137	0.067	0.571	0.283
Latency	20 (10–35)	30 (10–45)	15 (10–30)	15 (10–20)	**0.005**	**0.001**	0.169	0.073
Sleep time	7.0 (6.0–7.7)	6.9 (6.0–7.5)	7.0 (6.0–7.9)	6.8 (5.8–7.7)	0.651	0.907	0.465	0.537
Time in bed	8.0 (7.0–8.5)	8 (7–8.5)	8.0 (6.8–8.9)	7 (6–8)	**0.004**	**0.006**	**0.027**	0.859
Sleep efficiency (%)	87.5 (81.3–94.1)	87.5 (80.0–93.8)	88.2 (83.3–95.1)	96.7 (90.0–98.6)	**<0.001**	**<0.001**	**0.002**	0.399
PSQI	5 (4–7)	6 (5–8)	5 (4–6)	5 (3–7)	0.101	**0.014**	0.951	**0.027**
LPS	2 (0–4)	3 (0–5)	2 (0–3)	0 (0–3)	**0.004**	**0.001**	0.128	0.081
VAS	4 (0–5)	4 (0–6)	3 (0–5)	0 (0–2)	**<0.001**	**<0.001**	**0.002**	0.119

Statistically significant differences are in bold. Abbreviations: AC: active disease; AIS: Athens Insomnia Scale; BDI: Beck Depression Inventory; BMI: body mass index; ESS: Epworth sleepiness scale; HC: healthy control; IBD: inflammatory bowel diseases; LPS: Laitinen Pain Scale; n: number; NA: non-active disease; PSQI: Pittsburgh Sleep Quality Index; VAS: visual analogue scale.

**Table 2:** The results of sleep and mood related questionnaires and quantification of mRNA *SERT* and serotonin levels and SERT protein expression in participants with CD compared to UC.

	CD			UC			HC (n = 41)	*p* (CD-UC)	*p* (CD-HC)	*p* (UC-HC)	*p* (AC-NA in CD)	*p* (AC-NA in UC)
	All (n = 46)	AC (n = 27)	NA (n = 19)	All (n = 31)	AC (n = 18)	NA (n = 13)						
mRNA SERT	1.3 (0.4–2.2)	1.2 (0.7–2.2)	1.2 (0.3–2.8)	1.4 (0.7–2.1)	1.2 (0.5–2.2)	1.4 (0.8–1.6)	1.7 (1.0–2.3)	0.767	0.104	0.139	0.945	0.936
Serotonina	144.2 (96.0–180.4)	164.1 (120.8–221.0)	107.9 (51.5–145.7)	163.4 (100.0–208.7)	170.2 (94.9–298.8)	163.4 (124.0–187.2)	115.3 (76.6–160.5)	0.251	0.091	**0.008**	**0.002**	0.764
SERT	18.2 (±9.6)	19.7 (±10.1)	16.0 (±8.6)	19.2 (±7.4)	21.7 (±8.3)	15.7 (±4.3)	13.2 (±6.0)	0.628	**0.005**	**<0.001**	0.197	**0.025**
BDI	7 (4–10)	7 (5–10)	7 (3–11)	7 (3–11)	7.5 (5.0–11.0)	5 (3–9)	3 (1–8)	0.723	**0.008**	**0.040**	0.467	0.560
AIS total	6 (4–9)	7.3 (±4.2)	6.3 (±3.5)	4 (3–7)	5 (3–10)	4 (3–5)	4 (2–7)	0.055	**0.011**	0.631	0.386	0.210
ESS	6 (4–10)	6 (4–10)	5 (3–8)	7 (4–10)	7 (5–10)	6 (3–11)	6 (3–8)	0.606	0.289	0.116	0.494	0.469
Latency	30 (10–60)	30 (15–60)	20 (10–30)	15 (10–25)	17 (10–40)	15 (10–20)	15 (10–20)	0.054	**0.001**	0.200	0.133	0.402
Sleep time	7 (6–8)	7 (6–8)	6.8 (5.9–7.8)	6.6 (±1.1)	6.2 (±1.1)	7.1 (±1.0)	6.8 (5.8–7.8)	0.383	0.442	0.713	0.342	**0.021**
Time in bed	8.0 (7.0–8.5)	8.0 (7.5–9.0)	8.0 (6.5–8.5)	8.0 (7.0–8.5)	7.4 (±0.9)	8.2 (±1.4)	7 (6–8)	0.397	**0.004**	0.050	0.172	0.050
Sleep efficiency [%]	87.5 (83.3–93.8)	87.5 (84.2–93.8)	87.5 (83.3–94.1)	87.5 (80.0–95.8)	84.0 (75.0–95.8)	88.9 (85.7–95.8)	96.7 (90.0–98.6)	0.996	**<0.001**	**<0.001**	0.831	0.446
PSQI	5 (4–8)	6 (5–10)	5 (3–8)	5 (4–7)	6 (4–8)	5 (4–5)	5 (3–7)	0.405	0.099	0.279	0.113	0.139
LPS	3 (0–5)	3 (2–7)	3 (0–5)	2 (0–2)	2 (0–3)	2 (0–2)	0 (0–3)	**0.007**	**<0.001**	0.342	0.154	0.246
VAS	5 (0–6)	5 (2–7)	5 (0–5)	3 (0–4)	4 (0–5)	3 (0–3)	0 (0–2)	**0.031**	**<0.001**	**0.005**	0.302	0.109

Statistically significant differences are in bold. Abbreviations: AC: active; AIS: Athens Insomnia Scale; BDI: Beck Depression Inventory; ESS: Epworth sleepiness scale; HC: healthy control; LPS: Laitinen Pain Scale; n: number; NA: non-active disease; PSQI: Pittsburgh Sleep Quality Index; VAS: visual analogue scale.

**Table 3:** Correlations between the mRNA *SERT* and serotonin levels, SERT protein expression, patients’ ages, and the results of sleep- and mood-related questionnaires.

	Serotonin		SERT		mRNA *SERT*	
	IBD	HC	IBD	HC	IBD	HC
Serotonin	-	-	0.11; 0.357	**0.43; 0.005**	0.04; 0.760	−0.02; 0.895
SERT	0.11; 0.357	**0.43; 0.005**	-	-	−0.03; 0.786	0; 0.981
mRNA SERT	0.04; 0.760	−0.02; 0.895	−0.03; 0.786	0; 0.981	-	-
Age	−017; 0.146	−0.24; 0.137	0.06; 0.603	−0.19; 0.222	0.03; 0.768	0.2; 0.202
BMI	−015; 0.182	−0.01; 0.974	−0.03; 0.806	0.16; 0.332	0; 0.983	0.14; 0.379
HBI	**0.44; 0.002**	-	0.17; 0.253	-	−0.06; 0.711	-
PMS	0.05; 0.772	-	0.15; 0.41	-	0.15; 0.421	-

Statistically significant differences are in bold. Abbreviations: BMI: body mass index; HBI: Harvey–Bradshaw Index; HC: healthy control; IBD: inflammatory bowel disease; PMS: Partial Mayo Score.

**Table 4:** Associations between the serum level of mRNA *SERT* and serotonin levels, SERT protein expression, and the characteristics of patients with IBD, and the results of sleep- and mood-related questionnaires.

		mRNA *SERT* Expression		SERT (ng/mL)		Serotonin (ng/mL)	
Sex	Women	1.1 (0.4–1.5)	**0.011**	19.3 (±9.3)	0.406	163.4 (108.9–201.8)	0.068
	Men	1.8 (0.8–2.7)		17.7 (±8.0)		130.0 (94.9–173.6)	
Smoking	Yes	2.4 (1.1–4.1)	**0.049**	16.8 (±7.2)	0.468	144.3 (96.0–173.6)	0.585
	No	1.2 (0.5–2.0)		18.9 (±9.0)		148.6 (100.0–195.8)	
History of surgery	Yes	1.1 (0.6–2.0)	0.581	16.5 (±10.4)	0.190	161.8 (107.9–219.1)	0.401
	No	1.3 (0.5–2.2)		19.4 (±8.0)		144.1 (96.0–188.8)	
Other chronic diseases	Yes	1.1 (0.5–2.2)	0.516	18.7 (±9.5)	0.959	157.7 (105.4–205.0)	0.559
	No	1.3 (0.6–2.2)		18.6 (±8.6)		144.3 (96.1–188.8)	
BDI	>10 points	1.4 (0.5–1.9)	0.754	18.5 (±9.7)	0.950	145.7 (51.5–254.1)	0.864
	<11 points	1.2 (0.6–2.2)		18.6 (±8.5)		144.7 (105.0–188.8)	
AIS	>5 points	1.5 (0.7–2.7)	0.170	21.4 (±9.3)	**0.006**	145.1 (105.0–178.1)	0.744
	<6 points	1.1 (0.5–1.7)		16.0 (±7.5)		149.7 (98.1–197.0)	
ESS	>10	1.4 (1.1–1.6)	0.665	17.9 (±7.8)	0.716	142.8 (74.0–282.0)	0.811
	<11	1.2 (0.5–2.2)		18.8 (±9.0)		151.5 (100.3–188.8)	
PSQI	>5	1.2 (0.5–2.2)	0.923	19.1 (±10.6)	0.654	145.1 (68.0–254.1)	0.826
	<6	1.3 (0.7–2.2)		18.2 (±6.9)		145.0 (105.4–188.8)	
Glucocorticoids	Yes	1.2 (0.4–2.9)	0.751	18.3 (±10.6)	0.279	145.1 (68.0–254.1)	0.076
	No	1.3 (0.7–1.9)		18.2 (±6.9)		145.0 (105.4–188.8)	
Azathioprine	Yes	1.3 (0.4–2.5)	0.860	15.2 (±7.6)	0.448	145.7 (100.27–187.2)	0.677
	No	1.3 (0.7–2.0)		17.9 (±6.1)		144.7 (96.1–205.0)	

Statistically significant differences are in bold. Abbreviations: AIS: Athens Insomnia Scale; BDI: Beck Depression Inventory; ESS: Epworth sleepiness scale; PSQI: Pittsburgh Sleep Quality Index.

## Data Availability

The data presented in this study are available on request from the corresponding author.
